# Nuclear Magnetic Resonance (NMR)-Based Lipidomics Reveal the Association of Altered Red Blood Cell (RBC) Membrane Lipidome with the Presence and the Severity of Coronary Artery Stenosis

**DOI:** 10.3390/molecules30010036

**Published:** 2024-12-26

**Authors:** Ioanna A. Kastani, Paraskevi K. Soltani, Giannis G. Baltogiannis, Georgios A. Christou, Eleni T. Bairaktari, Christina E. Kostara

**Affiliations:** 1 Laboratory of Clinical Chemistry, Faculty of Medicine, School of Health Sciences, University of Ioannina, 45110 Ioannina, Greece; ioankast@gmail.com (I.A.K.); paraskevislt16@gmail.com (P.K.S.); ebairakt@uoi.gr (E.T.B.); 2 Private Practice, Ioannina & St. Luke’s Hospital Thessaloniki, 55236 Thessaloniki, Greece; yannibalt@hotmail.com; 3 First Department of Cardiology, Faculty of Medicine, School of Health Sciences, University of Ioannina, 45110 Ioannina, Greece; georgios.christou@yahoo.gr

**Keywords:** coronary heart disease, coronary artery stenosis, erythrocytes membrane, red blood cells, NMR spectroscopy, lipidomics, cholesterol, sphingomyelin, phospholipids, fatty acids

## Abstract

Coronary heart disease (CHD) is the leading cause of morbidity and mortality worldwide despite significant improvements in diagnostic modalities. Emerging evidence suggests that erythrocytes, or red blood cells (RBCs), are one of the most important contributors to the events implicated in atherosclerosis, although the molecular mechanisms behind it are under investigation. We used NMR-based lipidomic technology to investigate the RBC lipidome in patients with CHD compared to those with normal coronary arteries (NCAs), all angiographically documented, and its correlation with coronary artery stenosis. Targeted and untargeted lipidomic analysis revealed that CHD patients presented significant lipid alterations in the RBC membrane, characterized by higher cholesterol, sphingolipids, saturated and monounsaturated fatty acids, lower phospholipids (glycerophospholipids and ether glycerolipids), and unsaturated and polyunsaturated fatty acids. These aberrations gradually distinguish the three subgroups of patients with mild, moderate, and severe coronary stenosis, potentially indicating their non-negligible involvement in the onset and progression of atherosclerosis. The comprehensive analysis of RBC-membrane-derived lipids with omics approaches could unravel specific lipid abnormalities taking place at the silent subclinical stage of atherosclerosis and could have the potential to identify patients with subtle, but still proatherogenic, abnormalities that may confer a higher risk for the development of CHD.

## 1. Introduction

Coronary heart disease (CHD) is the leading cause of morbidity and mortality worldwide, despite significant improvements in diagnostic modalities and therapeutic strategies in recent years [1]. The dysregulation of lipid metabolism assessed by traditional serum lipid profile plays a well-documented role in the pathogenesis of CHD. Established traditional factors, including dyslipidemia, age, gender, smoking, and hypertension, have been implicated in the development of the disease and are the mainstay of cardiovascular (CV) risk assessment [2]. Because of the complex and multifactorial etiology of CHD, the aforementioned factors explain a large proportion of the attributable risk [3] but frequently fail to identify individuals at high CV risk [4]. In a recent study, a specific cluster of serum metabolites, rather than a single biomarker, was able to identify patients who developed coronary artery disease and those who did not, despite comparable high-CV-risk profiles [5]. Therefore, there is an urgent need to identify clinically applicable biomarkers that, alone or combined with the existing ones, could add to the predictive power of established risk factors [6,7] and also a need to reveal additional abnormal molecular mechanisms that may act independently of and/or complementary to those currently known in the pathogenesis of CHD.

In this context, erythrocytes, or red blood cells (RBCs), beyond their vital role in the gas transport between tissues, are involved in several additional biological processes in cardiovascular homeostasis and atherosclerosis. Emerging evidence regards RBCs as one of the most important contributors to the molecular events implicated in the formation of atheromatous core [8], the initiation and progression of atherosclerotic plaque [9,10], and atherothrombotic complications [11,12].

Studies have shown that RBCs undergo functional alterations and disturbances in their biophysical properties, contributing to changes in rheology and hemodynamics in atherosclerotic diseases and microvascular disorders such as diabetes [13,14]. Changes in RBCs’ morphodynamic features, inter alia aggregability and deformability, were observed in patients with different grades of coronary stenosis and were linked to an increased risk of acute coronary events [15]. The impairment of deformability may cause significant disturbances in micro- and macrocirculation, leading to increased flow resistance and blood viscosity. Increased RBC adhesion could initiate or aggravate plaque rupture and thrombus formation [12,16], and increased aggregation was directly associated with vascular complications [17].

In addition to their impact on blood flow regulation, data from histopathological studies revealed the presence of erythrocytes’ membranes in the necrotic lipid core of atherosclerotic plaques in the coronary [18] and systemic vasculature [19]. Erythrocyte-derived lipids, of which free cholesterol represents a significant proportion, rather than those derived from foam cells, are predominantly accumulated within atherosclerotic plaques [20]. Among lipid components, cholesterol has gained more clinical attention as multiple studies conducted by Tziakas et al. have shown that its content was higher in patients with acute coronary syndromes compared to those with stable coronary artery disease and also positively influenced the burden and clinical instability of the atherosclerotic plaque [21,22,23,24]. However, the entire lipid profile of RBC membranes, consisting of different lipid classes with unique and critical impacts on membrane structure, physico–chemical properties, and function, has not been fully investigated in patients with stable CHD or in the progression of CHD.

The evolving technology of lipidomics, a subfield of metabolomics, concerns the systemic identification and quantification of lipid entities in biological fluids, determining the individual’s physiological state and providing deep insight into the etiology and pathogenesis of perturbed lipid-homeostasis-related disease states [25]. Disturbances in the lipidome can be interrogated with the use of platforms consisting of high-field-resolution nuclear magnetic resonance (NMR) spectroscopy coupled with multivariate statistical methods. NMR spectroscopy is a powerful and reliable tool to provide structural elucidation and qualitative and quantitative analyses of lipid molecules in biological samples with high reproducibility [26]. The analysis of the complex lipidomic datasets generated by multivariate statistical analysis (MVA) represents a powerful exploratory tool for identifying causal links between biomarkers and disease phenotypes.

To the best of our knowledge, the novelty of the present study concerns the global characterization of the lipidome of RBC membranes in patients with CHD compared to those with normal coronary arteries (NCAs), all angiographically documented, using the emerging proton ^1^H NMR-based lipidomic technology, and the investigation of the association of the alterations occurring in RBC lipidome with the severity of coronary artery stenosis.

## 2. Results

### 2.1. ^1^H NMR Analysis of the RBC Lipid Extracts

**Figure 1:**
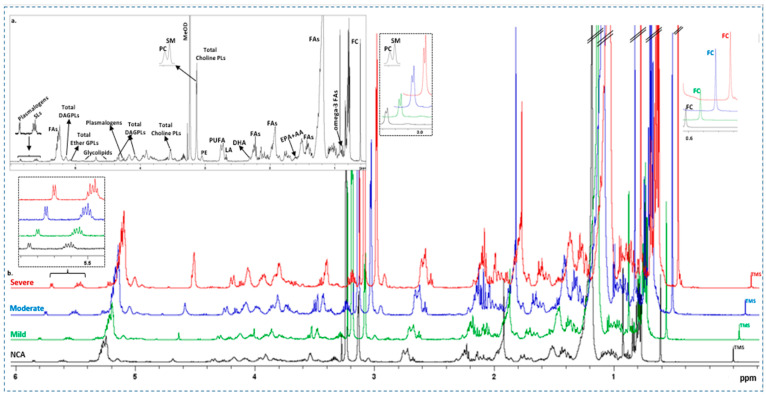
(**a**) ^1^H NMR spectrum of RBC membrane lipid extracts from a patient with NCA (black color). Peak assignments are summarized in Table 1. (**b**) ^1^H NMR spectra of RBC membrane lipid extracts from patients with NCA (black color) and patients with mild (green color), moderate (blue color), and severe (red color) coronary artery stenosis. Spectral intensity was normalized for the Tetramethylsilane (TMS) peak at 0.00 ppm.

Figure 1a shows a representative ^1^H NMR spectrum of RBC membrane lipid extract from a patient with NCA that contains signals attributed to protons of free cholesterol, headgroups, and backbones of phospholipids (glycerophospholipids and sphingolipids), glycerol backbone, and fatty acids—all of them being esterified and glycolipids (Table 1). In addition, typical ^1^H NMR spectra of the RBC membrane lipid extracts obtained from a patient with NCA and patients with mild, moderate, and severe coronary artery stenosis are presented in Figure 1b.

### 2.2. Characteristics of Study Population

Table 2 compares the main demographic, clinical, and biochemical data of the populations studied as defined below in the Section 4. Patients with CHD, overall and in subgroups, were selected with a similar age distribution and male prevalence and were well matched for serum lipid profile (total cholesterol, triglycerides, HDL-cholesterol, LDL-cholesterol, and non-HDL-cholesterol), apolipoprotein AI (apoAI) and apolipoprotein B (apoB) to minimize their confounding effect on the analysis.

### 2.3. RBC Lipidome Is Altered in Patients with Established CHD

We applied an independently untargeted and targeted lipidomic approach to identify differences in the lipid profiles of RBC membranes in patients with CHD and those with NCA, all angiographically determined, and for the quantitative alterations in the individual lipid classes.

**Figure 2:**
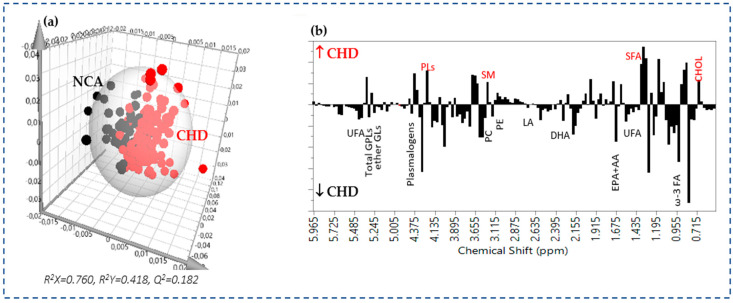
(**a**) OPLS-DA scores plot of the ^1^H NMR lipidomic data of RBC membrane lipid extracts from 121 patients with CHD (red circles) and 46 patients with NCA (black circles), (**b**) the corresponding OPLS-DA loading coefficient plot colored according to the correlation between the NMR lipidomic data and the group studied. Lipid constituents of RBC membranes presented in relatively higher levels in the CHD group deflect upwards and in relatively lower levels downwards.

For the untargeted multivariate analysis, the lipidomic data set (bins) derived from the 1D ^1^H NMR spectra of the lipid extracts of RBCs membranes from the 167 angiographically documented patients was as follows: 46 with NCA and 121 with CHD, further subdivided into three groups as described above: 48 with mild coronary artery stenosis, 36 with moderate, and 37 with severe coronary artery stenosis. RBC lipidomic data were initially explored by the unsupervised principal component analysis (PCA) in order to check their consistency and quality. The application of a supervised OPLS-DA analysis showed discrimination between NCA and CHD patients with good goodness of fit and predictive power (R^2^X = 0.760, R^2^Y = 0.418, Q^2^ = 0.182) and a minimal area of overlap (Figure 2a), indicating that the RBC lipid profiling was significantly different between the two study populations. NCA patients are placed on the left half of the plot, whereas CHD patients are on the right half. The model’s statistical significance was assessed by a *p*-value *<* 0.01 calculated by analysis of variance (CV ANOVA).

The analysis of the loading coefficient plot, which means the relative significance of each lipid constituent in the groups’ separation (Figure 2b), revealed that the determining lipid entities were primarily the lower content of omega-3 fatty acids, docosahexaenoic acid (DHA), the sum of eicosapentaenoic and arachidonic acids (EPA + AA) in the RBCs of CHD patients and the higher content of saturated fatty acids (SFA), cholesterol, and sphingomyelin (SM). Furthermore, RBCs’ membranes in CHD patients were found to be depleted in phosphatidylcholine (PC), glycerophospholipids (GPLs), phosphatidylethanolamine (PE), plasmalogens, and ether glycerolipids (ether GLs).

**Figure 3:**
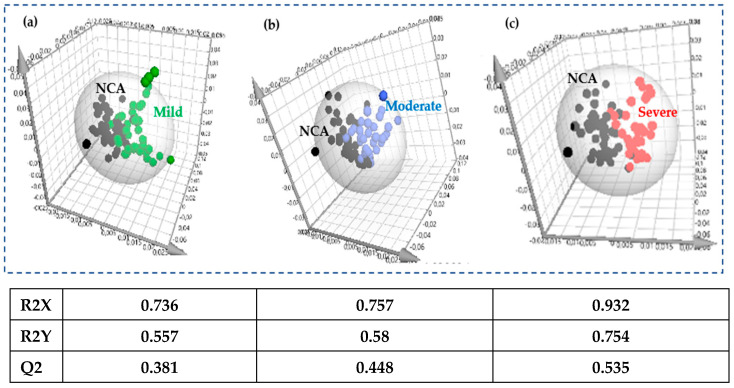
OPLS-DA scores plots of the untargeted analysis of the 46 patients with NCA (black circles) and (**a**) the 48 patients with mild coronary stenosis (green circles), (**b**) the 36 patients with moderate coronary stenosis (blue circles), and (**c**) the 37 patients with severe coronary stenosis (red circles). The ellipses surrounding the samples denote each group.

In the OPLS-DA comparison of the NCA patients with the three CHD subgroups, mild, moderate, and severe (Figure 3a–c), the more severe the coronary artery stenosis is assessed, the more the values of the parameters R^2^X and R^2^Y and of the predictive capability Q^2^ are increased, with *p*-value < 0.01 in all comparisons. These results are also reflected in the visual inspection of the graphs, where a gradual decrease in the overlap between the pairs can be seen.

Thereafter, we independently performed a quantitative targeted analysis of individual lipid constituents. As described below, the results in the targeted analysis are nearly consistent with those found in the untargeted multivariate analysis. Also, the % lipid composition of RBC membrane in our study population determined by NMR spectroscopy was nearly identical to literature values obtained from studies in which patients with CHD were enrolled [27,28,29]. Table 3 illustrates the percentages of the RBC membrane lipid molecules (free cholesterol and phospholipid classes (glycerophospholipids, ether glycerolipids, and sphingolipids)), as well as their ratios in the two studied groups. Compared to NCA patients, in CHD patients, the percentage of cholesterol was increased, whereas that of total phospholipids (Pls) was decreased, mainly due to the lower percentages of both GPLs and ether GLs (Table 3). The percentages of GPLs that were determined by the NMR spectrum such as PC and PE, as well as the rest of GPLs, were significantly lower in RBC membranes isolated from CHD patients compared to those with NCA. Also, the percentages of total ether GLs, plasmalogens, and the rest ether lipids were lower in CHD patients compared to NCA (Table 3). Notably, the contents of total sphingolipids (SLs), SM, and the rest sphingolipids, mainly attributed to ceramide, were significantly higher in CHD compared to NCA patients. Finally, glycolipids content did not change significantly between the two groups. The aforementioned changes resulted in alterations in molar lipid ratios that could reflect membrane fluidity, including an increase in the ratios of cholesterol to total PLs, SM to PC, cholesterol to PC, cholesterol to PE, and PE to PC in CHD patients compared to those with NCA, and a decrease in the ratios of cholesterol to SM and PE to SM, respectively (Table 3).

Table 4 summarizes the percentages of fatty acids (saturated and unsaturated fatty acids, mono- and polyunsaturated fatty acids, and individual polyunsaturated fatty acids) in the two groups studied. CHD patients presented marked changes in the fatty acid pattern of RBC membrane PLs compared to NCA (Table 4). In particular, in CHD patients, the erythrocyte membrane was enriched in SFAs and depleted in unsaturated (UFA) ones, mainly due to polyunsaturated fatty acids (PUFA), whereas monounsaturated fatty acid (MUFA) content was significantly higher. The percentages of individual PUFA, e.g., linoleic acid (LA), DHA, and the sum of EPA + AA were significantly lower in CHD than in NCA patients (Table 4). These changes in the fatty acid pattern resulted in significantly higher ratios of SFA to UFA and of SFA to PUFA in CHD patients compared to the NCA group.

### 2.4. Alterations Occurring in RBC Lipidomes Follow the Degree of Progression of Coronary Arteries Stenosis

**Figure 4:**
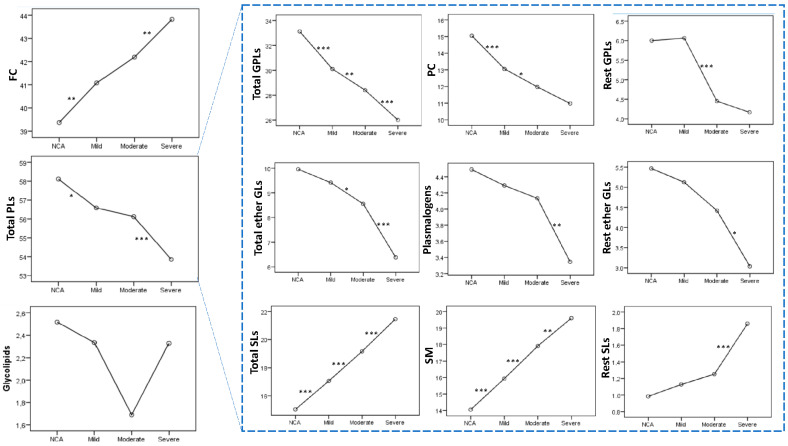
The % content of membrane lipids in NCA patients and patients with mild, moderate, and severe coronary artery stenosis. * *p* < 0.05, ** *p* < 0.01, *** *p* < 0.001 in the pairwise comparison performed using one-way analysis of variance (ANOVA).

Furthermore, we independently performed both targeted and untargeted lipidomic analysis to investigate whether the changes occurring in RBC lipidomes follow the progression of CHD. Erythrocyte membrane cholesterol content was gradually increased from he NCA group to the CHD subgroups (mild, moderate and severe) following the degree of coronary arteries stenosis (39.37 ± 2.49, 41.08 ± 2.33, 42.19 ± 2.28 and 43.82 ± 3.15, Figure 4), whereas PLs’ content was gradually decreased in the same direction (58.11 ± 2.70, 56.59 ± 2.94, 56.12 ± 2.69 and 53.85 ± 3.35, Figure 4). Glycolipid content was progressively decreased from NCA to mild and then to moderate CHD patients. However, compared to the moderate group, patients with severe disease presented with a higher glycolipid content.

As seen in Figure 4, a gradual decrease from the NCA group to the three CHD subgroups, following the severity of coronary artery stenosis, was also presented for the percentages of total GPLs (33.12 ± 2.88, 30.11 ± 2.23, 28.40 ± 3.35, 26.01 ± 1.82), PC (15.05 ± 2.64, 13.05 ± 2.28, 11.97 ± 2.45, 10.98 ± 1.64), total ether GLs (9.96 ± 2.03, 9.41 ± 2.24, 8.55 ± 1.45, 6.38 ± 1.87), plasmalogens lipids (4.49 ± 1.29, 4.29 ± 1.09, 4.13 ± 1.03, 3.35 ± 1.30) and of the rest ether GLs (5.47 ± 2.09, 5.12 ± 2.06, 4.42 ± 1.28, 3.04 ± 1.85). The percentage of the other GPLs was similar in patients with NCA and in those at the onset of disease (mild CHD patients) (6.00 ± 1.73 and 6.07 ± 1.71) and was decreased in the moderate and even more in the severe CHD group (4.45 ± 1.11 and 4.17 ± 1.31, respectively). The changes in PE did not follow the progression of disease (12.08 ± 1.21 (NCA patients), 10.99 ± 1.51 (mild CHD), 11.97 ± 1.83 (moderate CHD), and 10.86 ± 1.72 (severe CHD)). It is worth noting that the percentages of total SLs (15.03 ± 2.24, 17.06 ± 1.84, 19.16 ± 2.69, 21.46 ± 3.01), SM (14.05 ± 2.31, 15.93 ± 1.92, 17.91 ± 2.68, 19.60 ± 3.07), and those of the other SLs (0.98 ± 0.46, 1.13 ± 0.71, 1.25 ± 0.87, 1.86 ± 1.11) were gradually increased from NCA patients to mild, moderate, and then severe CHD patients (Figure 4).

**Figure 5:**
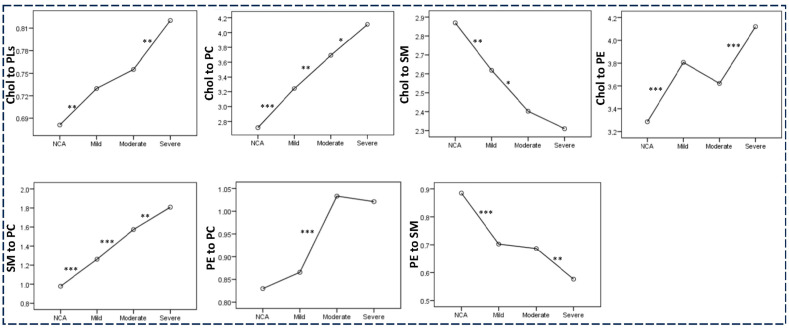
Membrane lipid ratios in NCA patients and patients with mild, moderate, and severe coronary artery stenosis. * *p* < 0.05, ** *p* < 0.01, *** *p* < 0.001 in the pairwise comparison performed using one-way analysis of variance (ANOVA).

The qualitative and quantitative aberrations in RBC membrane lipids described above resulted in significant changes in their molar ratios. As seen in Figure 5, a progressive decrease from NCA to mild, moderate, and then severe CHD patients was observed for the molar ratios of cholesterol to SM (2.87 ± 0.45, 2.62 ± 0.37, 2.40 ± 0.35, 2.31 ± 0.53) and of PE to SM (0.88 ± 0.18, 0.70 ± 0.14, 0.68 ± 0.16, 0.58 ± 0.16), whereas the ratios of cholesterol to PLs (0.68 ± 0.07, 0.73 ± 0.08, 0.76 ± 0.07, 0.82 ± 0.11), cholesterol to PC (2.72 ± 0.62, 3.24 ± 0.60, 3.69 ± 0.89, 4.11 ± 0.87), and SM to PC (0.98 ± 0.30, 1.26 ± 0.29, 1.57 ± 0.45, 1.81 ± 0.30) gradually increased in the same direction. The ratio of PE to PC increased from NCA patients to the mild and then to the moderate CHD group (0.83 ± 0.18, 0.87 ± 0.18, 1.03 ± 0.22 Figure 5). Compared to the moderate group, the severe one presented a slight but not statistically significant decrease in the ratio PE to PC (1.02 ± 0.27 vs. 1.03 ± 0.22, *p* > 0.05). The ratio of cholesterol to PE was significantly higher in mild CHD compared to NCA patients and in severe compared to moderate CHD. Patients with moderate CHD presented with a lower cholesterol-to-PE ratio compared to mild patients.

**Figure 6:**
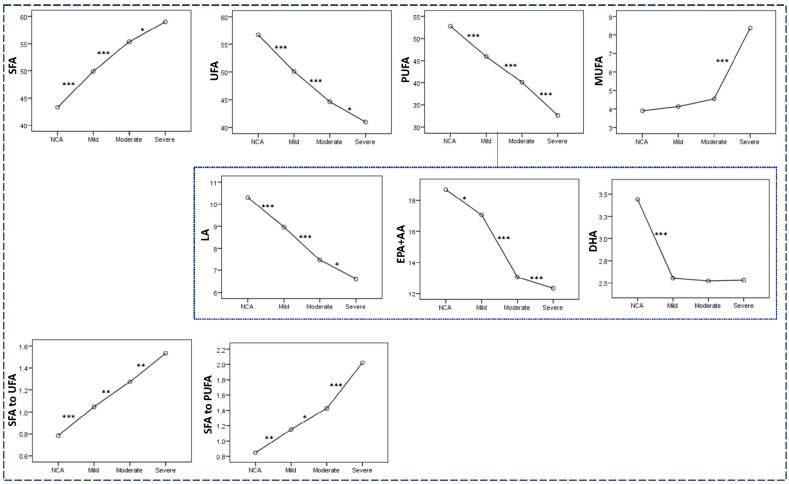
The % content of membrane fatty acids in NCA patients and patients with mild, moderate, and severe coronary artery stenosis. * *p* < 0.05, ** *p* < 0.01, *** *p* < 0.001 in the pairwise comparison performed using one-way analysis of variance (ANOVA).

The fatty acid pattern of the RBC membrane phospholipids was altered following the progression of coronary artery stenosis (Figure 6). More specifically, the percentages of SFA (43.31 ± 6.22, 49.90 ± 7.31, 55.36 ± 5.72, 59.02 ± 7.62) and MUFA (3.90 ± 1.73, 4.13 ± 1.56, 4.54 ± 1.74, 8.37 ± 2.69) were gradually increased from the NCA group to patients with mild, moderate, and severe CHD. On the other hand, the percentages of UFA (56.69 ± 6.22, 50.10 ± 7.31, 44.64 ± 5.72, 40.98 ± 7.62), total PUFA (52.79 ± 6.59, 45.97 ± 7.23, 40.09 ± 5.81, 32.62 ± 8.71), and individual PUFAs such as LA (10.31 ± 2.15, 8.96 ± 1.78, 7.46 ± 1.48, 6.61 ± 1.53) and the sum of EPA + AA (18.70 ± 4.17, 17.07 ± 4.17, 13.05 ± 3.68, 12.33 ± 3.14) were progressively decreased in the same direction (Figure 6). Of note, DHA content remained unchanged among the three CHD subgroups [2.55 ± 0.82 (mild coronary stenosis), 2.52 ± 0.40 (moderate coronary stenosis), and 2.53 ± 0.55 (severe coronary stenosis)] and markedly lower compared to that in NCA (3.44 ± 0.83). The aforementioned changes resulted in a gradual increase in the ratios of SFA to UFA (0.79 ± 0.20, 1.05 ± 0.37, 1.27 ± 0.28, 1.53 ± 0.52, respectively) and sSFA to PUFA (0.85 ± 0.23, 1.15 ± 0.45, 1.43 ± 0.33, 2.02 ± 0.85, respectively) from NCA to all CHD subgroups following the severity of the disease (Figure 6).

**Figure 7:**
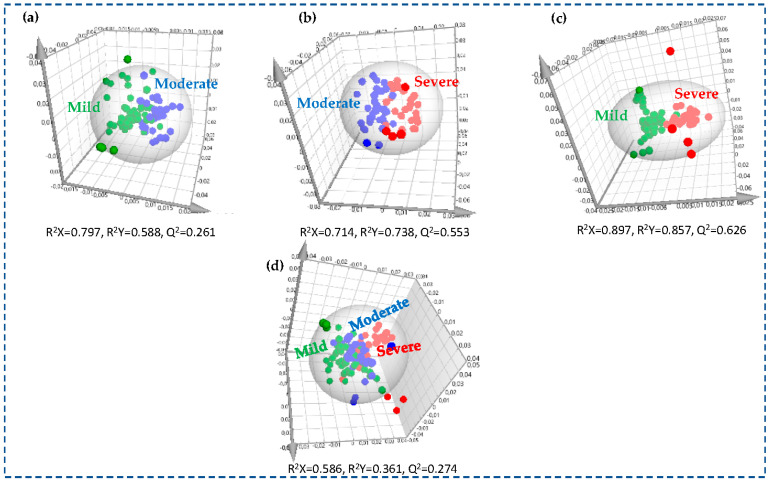
OPLS-DA scores plot (t1 vs. t2) of the RBC membrane’ lipidomic targeted analysis of the three patients’ subgroups: (**a**) 48 patients with mild coronary stenosis (green circles) and 36 patients with moderate coronary stenosis (blue circles); (**b**) 36 patients with moderate coronary stenosis (blue circles) and 37 patients with severe coronary stenosis (red circles); (**c**) 48 patients with coronary stenosis (green circles) and 37 patients with severe coronary stenosis (red circles) (**d**) 48 patients with mild coronary stenosis (green circles), 36 patients with moderate coronary stenosis (blue circles), and 37 patients with severe coronary stenosis (red circles). The ellipses surrounding the samples denote each group.

Finally, the pairwise comparisons of the three CHD subgroups (mild vs. moderate, moderate vs. severe, and mild vs. severe) using OPLS-DA models created with untargeted analysis are shown in Figure 7a–c. The quality parameters of the above-described progressive qualitative and quantitative alterations in membrane lipids observed in CHD subgroups were higher for the moderate–severe and mild–severe pairs than in the untargeted analysis, allowing an effective visualization of the progression (Figure 7d), suggesting a relatively high impact of the severity of coronary arteries stenosis on the RBC lipidome. The separation among three subgroups was assessed by the following quality parameters of the model: R^2^X = 0.586, R^2^Y = 0.361, and Q^2^Y = 0.274; the CV-ANOVA *p*-value was <0.001.

## 3. Discussion

In the present study, we used a ^1^H NMR-based lipidomic approach to investigate the alterations in lipid composition of RBC membranes in CHD patients compared to those with NCA and whether these changes were associated with the angiographically assessed coronary artery stenosis. The targeted and untargeted lipidomic analysis revealed that patients with the onset of CHD and at any disease stage presented significant alterations in the RBC lipidome compared to NCA patients. These aberrations were able to gradually distinguish the three angiographically documented CHD patients’ groups, potentially indicating their nonnegligible contribution to the onset and progression of atherosclerosis.

The fatty acid pattern expresses the qualitative and quantitative dynamic balance between de novo metabolic pathways and nutrition. Especially for erythrocytes, the fatty acid composition of membrane phospholipids is considered a better long-term marker of dietary lipid changes because of their slower turnover rate than that of plasma lipids [30]. A limited number of studies have investigated the fatty acid composition of erythrocyte membranes in patients with CHD [31,32]. However, the association between the changes in fatty acid patterns and the severity of CHD has not been investigated in depth.

In our study, both untargeted and targeted analysis showed that the fatty acid pattern of erythrocyte membrane phospholipids was significantly different in patients with CHD compared to those with NCA. Of note, the observed aberrations were associated with the severity of coronary artery stenosis. Specifically, CHD patients showed an increase in the percentages of SFA and MUFA and a decrease in the percentages of UFA, total PUFA, and individual PUFAs, e.g., LA, the sum of EPA + AA, and DHA, resulting in an increase in the SFA/UFA and SFA/PUFA ratios.

Studies have shown that the quality of dietary fatty acid patterns determines the risk of developing CHD and stroke [33,34]. SFAs are the main fatty acid component of cell membrane lipids. Lauric acid, myristic acid, palmitic acid, and stearic, acid, which represent the major SFAs, were associated with an increased risk of CHD, whereas PUFA content had an inverse relationship with the incidence of CHD [35]. The replacement of SFAs with PUFAs was associated with a lower CHD risk [36,37]. Thus, the earlier detection of changes in dietary-fat-associated CHD risk would be beneficial for preventing CHD development and progression.

MUFA content was significantly higher in CHD patients compared to those with NCA and was gradually increased among CHD groups following the progression of disease. A large prospective cohort revealed that high serum MUFA levels were considered an independent biomarker for future cardiovascular events [38]. The conversion of SFAs into MUFAs is catalyzed by a delta-9-desaturase, the stearoyl-CoA desaturase-1 (SCD1). SCD1 has been involved in the development and progression of cardiovascular disease and was positively correlated with its mortality [39] and hence is thought to be a promising target for treatment.

The erythrocyte membrane LA content, one of the omega-6 PUFAs, was significantly decreased in CHD patients compared to NCA and decreased gradually among CHD groups following the severity of coronary arteries stenosis, a finding that is in agreement with previous studies [29,40]. Namazi G et al. [29,40] have shown that the erythrocyte LA levels were inversely correlated with the CAD scores (the CAD score was calculated as follows: the number of major coronary arteries, including the left anterior descending, circumflex, and right coronary arteries, with no luminal stenosis to complete luminal stenosis assigned scores between 0 and 3, respectively) after adjustment for multiple parameters. Higher dietary intake or circulating LA levels were significantly associated with a decreased risk of CV events [41,42,43] and deaths [44]; however, the exact molecular mechanisms have not been elucidated. Given that LA is an essential, exclusively diet-derived fatty acid, inadequate intake could contribute to the onset and progression of CHD in the present study.

Untargeted analysis showed that long-chain omega-3 PUFA content, determined as the sum of EPA plus DHA to total membrane lipid content, was lower in CHD patients compared to NCA and contributed to their separation, with the highest coefficient among the lipid entities. The RBC levels of EPA plus DHA were associated with the early onset of coronary atherosclerosis [45] and inversely associated with total mortality rates from cardiovascular disease [46]. In the last decade, the percentage of EPA plus DHA of total fatty acids, named the omega-3 index, has been proposed as a risk factor for death from cardiovascular disease (CVD) [47,48]. This fatty acid metric was inversely associated with the risk for acute coronary syndromes [49]. The molecular mechanisms explaining the clinical benefits offered by omega-3 fatty acids may be mediated via their involvement in plaque-stability enhancement [50] and cell membrane stabilization, as well as via their antithrombotic, anti-inflammatory, or antiarrhythmic properties [51,52]. Moreover, it has been shown that omega-3 fatty acids may modulate atherogenesis by affecting the processes of endothelial activation and cause a beneficial shift in the eicosanoid system decreasing the platelet aggregation and blood pressure [53,54].

The accumulation of lysed erythrocyte membranes in atherosclerotic plaques and the subsequent processes occurring within create an atherogenic milieu that contributes to the progression of atherosclerosis [55]. It has been suggested that erythrocyte-derived free cholesterol rather than that derived from foam cells predominantly accumulates within atherosclerotic plaques through repeated intraplaque hemorrhage occurring over years [18,20,56]. The continuous deposition of cholesterol evokes the progression of the atherosclerotic process [57], contributes to the expansion of the necrotic lipid core, and also promotes plaque vulnerability and instability [19,56,58].

In our study, erythrocyte membrane-free cholesterol content was significantly higher in CHD patients compared to NCA, which is in agreement with previous findings [59]. This change was observed notwithstanding serum cholesterol levels being similar among the study population groups, possibly suggesting that the mechanisms responsible for cholesterol uptake and/or efflux by the erythrocytes are more complicated. Clinical studies have shown that erythrocyte cholesterol levels were higher in patients with acute coronary syndrome compared to those with stable coronary artery disease [24,60] and were strongly associated with clinical instability in coronary artery disease patients in an independent and incremental manner [22,23]. Interestingly, cholesterol levels gradually increased among our CHD groups following the progression of angiographically assessed coronary artery stenosis. However, the findings concerning the intricate relationship between erythrocyte cholesterol content and the angiographic degree of coronary atherosclerosis are contradictory [24,61]; thus, a large-scale clinical investigation is warranted.

Erythrocytes are important determinants of blood flow dynamics via their hemorheological properties, e.g., shape deformability and adhesion-aggregation, which are governed by membranes’ elastic properties. Increased erythrocyte aggregation may affect vascular function through its effects on the distribution of flowing erythrocytes, whereas decreased deformability may result in reduced blood flow in areas where the blood flow is already low and/or where there is advanced coronary artery narrowing. Abnormalities in these rheological properties may underlie the defects that favor the development and progression of atherosclerotic coronary artery disease [13,62,63]. Recently, the morphodynamic characteristics of erythrocytes have been associated with plaque instability in high-risk nonobstructive coronary artery disease patients, suggesting the use of these blood cell features in the identification of high-risk patients in the absence of severe coronary stenosis [15].

Erythrocyte deformability depends on its geometry, internal cytoplasmic viscosity, membrane fluidity, and flexibility, which are strongly determined by its lipid composition [64]. Accumulating data support the notion that alterations in RBCs’ membrane lipid composition promote modifications in both the ultrastructure and rheological properties, leading to morphological abnormalities and hemorheological perturbations [65], which contribute to partly impaired functional status, as well as increased vascular resistance and enhanced interaction of these cells with the vascular wall. In our study, concurrent with the aforementioned changes in the fatty acid pattern and cholesterol content that both induce membrane stiffness and reduce bilayer fluidity [66], alterations in phospholipid classes and their proportions also characterized the initial stages of the disease and its progression.

The molecular ratio of cholesterol to total PLs was statistically significantly higher in CHD patients compared to NCA and was gradually increased among CHD groups following the severity of disease. Changes in this ratio lead to morphologically abnormal erythrocytes with a decreased life span. In addition, this ratio has been proposed as a qualitative parameter for the correlation with the lipid microviscosity and rheological properties of RBCs [67].

Phosphatidylcholine and sphingomyelin, which are the most abundant choline-containing phospholipids dominating the outer leaflet of RBC membranes, are highly dissimilar, corresponding to their influence on RBC membrane fluidity [68]. SM increases the rigidity of the membrane lipid bilayer, whereas PC maintain the bilayer in a more fluid state [69,70]. The percentage of SM was significantly higher in CHD patients than in NCA, whereas that of PC was lower, resulting in a statistically significant higher ratio of SM to PC in these patients. This trend of change for PC and SM (decrease and increase, respectively) was also gradually observed from mild to moderate and then to severe CHD patients, potentially indicating that the cell membrane becomes stiffer and less fluid with the severity of coronary artery stenosis.

Erythrocyte membrane SM acts as a molecular “trap” of free cholesterol within the membrane, regulating the amount that is absorbed [71]. The kinetics and the mechanism of the bidirectional flux of free cholesterol between erythrocytes and plasma lipoproteins were closely affected by plasma SM levels and the ratio of SM to PC [72]. It has been reported that SM depletion resulted in a loss of cholesterol from the cell membrane [73]. Cholesterol content was positively correlated with that of SM and presented a significant and independent association with the presence of acute coronary syndrome, suggesting a possible link between SM levels and clinical instability, which may, in turn, reflect the underlying atheromatous plaque instability [74]. In our study, cholesterol content was increased concurrently with the increase in SM in all CHD patients, and progressively from the mild to moderate and then to the severe CHD group, a finding that is consistent with the role of SM as a molecular attraction for cholesterol and potentially as an atherogenic agent.

CHD patients presented with a higher percentage of the remaining sphingolipids, mainly attributed to ceramide, compared to the NCA group. The severe CHD group was presented with the highest content of ceramide compared to other groups. High ceramide levels in erythrocyte membrane were found to disrupt the membrane–cytoskeleton interaction and membrane integrity, leading to vesiculation, reduced deformability, and finally a loss of red blood cell content [75]. Given that erythrocytes are incapable of performing de novo biosynthesis due to the lack of organelles, increased membrane ceramide possibly reflects high serum ceramide levels. Serum ceramide concentrations have been shown to be linked with an increased risk of major adverse cardiovascular events in patients with and without coronary artery disease [76,77].

Plasmalogens, the most common ether-linked phospholipids, contain a vinyl ether moiety at the sn-1 position of their glycerol backbone and an ester-linked fatty acid at the sn-2 position [78]. This vinyl ether linkage is more preferentially affected by reactive oxygen species than fatty acyl chain analogs, indicating a possible “scavenger” function activity of plasmalogens in cell membranes and lipoproteins [79]. The low percentages of total ether glycerophospholipids, plasmalogens, and the other ether glycerophospholipids in the total CHD patients compared to NCA, as well as progressively from mild to moderate and then to severe CHD patients, possibly indicate an enhanced susceptibility of erythrocytes to metabolic and oxidative stress in these patients.

## 4. Materials and Methods

### 4.1. Subjects

A total of 121 patients with a confirmed diagnosis of acute coronary syndrome, without persistent elevation of the ST segment in the electrocardiograph (NSTE-ACS) and who angiographically demonstrated one- (mild), two- (moderate), or three- (severe) vessel disease (defined as the presence of ≥50% diameter luminal narrowing in one, two, or three of the major epicardial vessel systems), were admitted to the Coronary Care Unit of the University Hospital of Ioannina and participated in the study. Forty-six consecutive patients, admitted to the hospital because of atypical episodes of chest pain without any increase in biochemical markers, who met the inclusion and exclusion criteria of the study (as defined below), had angiographically normal coronary arteries (NCA). All patients underwent diagnostic coronary angiography within 7–9 days after the onset of the symptoms.

The diagnosis of NSTE-ACS was based on the criteria proposed by the European Society of Cardiology [80]. Patients who did not meet the criteria for NSTE-ACS after the initial evaluation were excluded from the study. Patients with a family history of premature cardiovascular disease, chronic renal disease, hepatic function impairment, chronic obstructive pulmonary disease, overt hyper-/hypothyroidism, or rheumatic diseases; patients on lipid-lowering drugs such as statins; patients on blood-pressure-lowering drugs that affect lipid metabolism (such as diuretics or beta blockers); and patients on lipid-lowering drugs were excluded from the study. For all participants in the study, demographic characteristics, smoking habits, body mass index (BMI), and personal history with regard to the presence of hypertension were recorded. All groups were matched for age, gender, and serum lipid profile (total, LDL-, non-HDL- and HDL-cholesterol, triglycerides), apoAI, and apoB to minimize the confounding effect of these parameters on the data analysis (Table 1). 

### 4.2. Sample Collection

Fasting venous blood samples were obtained in the morning before angiography and within the first 12 h after the onset of symptoms and were placed into vacutainer tubes for the isolation of serum. The serum was separated by centrifugation at 1500× *g* for 15 min and was used for the determination of the conventional biochemical parameters. In addition to the blood sample collected for biochemical analysis, a volume of 4 mL of venous blood was collected for the RBCs’ lipidomic analysis in a separate EDTA-containing tube (the EDTA concentration was 1.8 mg per ml of complete blood). The sample was immediately centrifuged at 1500× *g* for 10 min, allowing the separation of plasma and RBCs. After their isolation, RBCs were stored for a maximum of one month at −80 °C until lipid extraction.

### 4.3. Ethics Statement

The collection of the samples from all participants was conducted in accordance with the guidelines of the Ethics Committee of the University Hospital of Ioannina. Written consent was obtained from each participant.

### 4.4. Determination of Biochemical Parameters in Serum Samples

Clinical chemistry parameters were measured on an AU5800 Clinical Chemistry analyzer (Beckman Coulter, Hamburg, Germany) with standard procedures using enzymatic colorimetric methods. For the determination of total cholesterol, the enzymes cholesterol esterase and cholesterol oxidase were combined into a single enzymatic reagent, whereas for the determination of triglycerides, the enzymes glycerol kinase, glycerol phosphate oxidase, and peroxidase were combined. HDL-cholesterol was determined by a direct assay. LDL-cholesterol was calculated by the Friedewald formula, and non-HDL-cholesterol was calculated by the equation non-HDL-cholesterol = total cholesterol minus HDL-cholesterol. Serum apoAI and apoB were measured by immunonephelometry on a BN ProSpec System (Siemens, Marburg, Germany).

### 4.5. Isolation and Lipid Extraction of RBC Membranes

The lipid content of RBC membranes was extracted as described by Adosraku et al. [27], as described previously [81].

### 4.6. ^1^H NMR Spectroscopy

The dried lipid extracts of RBC membranes were redissolved in a 500 μL mixture of deuterated methanol/chloroform (2:1, *v*/*v*). All ^1^H NMR experiments were carried out on a Bruker Avance DRX NMR spectrometer (Bruker, Bremen, Germany) (NMR Center, University of Ioannina) operating at 500 MHz (proton resonance frequency). The temperature was kept constant at 298 K throughout the NMR experiments. A standard Bruker “zgpr” pulse program that applies a presaturation pulse sequence for the suppression of the residual water signal at about 4.75 ppm during the relaxation delay was used. Spectra were acquired in the Fourier transform (FT) mode with 32 K data points, 128 free induction decays (FID), 90° pulses, a relaxation delay of 4 s, and spectral widths of 5000 Hz. All FIDs were multiplied by an exponential weighting function corresponding to a 0.3 Hz line-broadening factor before FT. The acquired ^1^H NMR spectra were manually corrected for phase and baseline distortions and referenced to the methanol residual peak using TopSpin 2.1 (Bruker Biospin Ltd., Ettlingen, Germany). The quantification of the lipids was made as described previously [81]. The quantification of the membrane lipids was based on the integration of selected signals in the proton NMR spectrum, as listed in Table 1 [81], corrected for the number of protons, and then normalized with respect to the signal area from the cholesterol C18 methyl group at 0.68 ppm. The lipid composition of the RBC membrane was expressed as percentages of the total lipids of RBCs.

### 4.7. Statistical Analysis of Data

#### 4.7.1. Univariate Analysis

All data were expressed as the mean value ± SD. Group comparisons were performed using a one-way analysis of variance (ANOVA), followed by a least-significant-difference (LSD) test for pairwise comparisons. A *p*-value < 0.05 was considered to indicate statistical significance.

#### 4.7.2. Multivariate Analysis

Unsupervised and supervised multivariate techniques, principal component analysis (PCA), and orthogonal projections to latent structures discriminant analysis (OPLS-DA) were used to construct pattern-recognition models to extract specific lipid signatures of the RBC membranes associated with the onset and the progression of coronary artery stenosis. The OPLS-DA scores’ plot was used to reveal observations lying outside the 0.95 Hotteling’s T2 ellipse and to detect any grouping trend or separation, whereas the OPLS-DA loading coefficient plot was used to show the contribution of all NMR spectral regions or variables (corresponding to lipid constituents) to the grouping trend or the separation seen in the plot of scores. The performance of the model was assessed by goodness-of-fit parameters R^2^ (R^2^X and R^2^Y) and Q^2^, related to the explained and predicted variance calculated through 7-fold cross-validation, respectively. In addition, a cross-validated analysis of variance (CV-ANOVA) was also used to evaluate the statistical significance of the resulting OPLS-DA model. When the calculated CV-ANOVA *p* value is <0.5, the model is considered reliable.

## 5. Conclusions

The comprehensive analysis of erythrocyte membrane-derived lipids with omics approaches could unravel specific lipid abnormalities taking place in the silent subclinical stage of CHD and worsening with the progression of the disease, and they have the potential to identify patients with subtle proatherogenic abnormalities that may confer higher risk for the development of CHD.

In our study, the ^1^H NMR-based lipidomic analysis revealed that the onset and progression of CHD were characterized by similar and gradual disturbances in erythrocyte membrane lipid profiling, as illustrated by the abnormal content of cholesterol, phospholipids, and fatty acid patterns. These alterations may synergistically modify the functions and biophysical properties of erythrocytes, reinforcing their shift from a friendly to a potent atherogenic stimulus of the cellular and molecular mechanisms implicated in CHD pathogenesis. This knowledge could pave the way to potential therapeutical strategies targeting signaling in erythrocytes to prevent or reduce CHD incidence finding their niche in clinical practice.

## Figures and Tables

**Table 1:** Protons and Chemical Shift (in ppm) for the lipid constituents and headgroups identified in RBC lipid extract by NMR and selected signals for Quantification.

Lipid Constituents and Headgroups	^1^H NMR Signal Assignment	Chemical Shift (ppm)	Quantification of Lipids from Selected Well-Resolved NMR Signals
Cholesterol molecule	C_18_H_3_	0.68	Total Cholesterol, FC
	C_26_H_3_, C_27_H_3_, C_21_H	0.87	
	C_19_H_3_	1.00	
	C_3_H	3.40	
	C_6_H	5.36	
Glycerol backbone	C_1_H^u^ and C_3_H^u^ of glycerol backbone of DAGPLs	4.16	
	-OCH=CHCH_2_	4.32	PLA (ether GPLs)
	C_1_H^d^ and C_3_H^d^ of glycerol backbone of DAGPLs	4.40	
	C_2_H of glycerol backbone in ether glycerophospholipids	5.15	Ether GPLs
	C_2_H of glycerol backbone in total DAGPLs	5.18	Total Diacyl glycerophospholipids (DAGPLs)
Sphingosine moiety	-CH_2_-CH=CHCHOH	5.40	
	-CH_2_-CH=CHCHOH	5.70	Total SLs
Head-group & substituent	-CH_2_-CH_2_-N^+^(CH_3_)_3_ (choline)	3.20	Total choline-containing PLs (PC, SM)
	-CH_2_-CH_2_-N^+^(CH_3_)_3_	3.59	
	-CH_2_-CH_2_-N^+^(CH_3_)_3_	4.24	
	-CH_2_-CH_2_-NH_3_^+^ (ethanolamine)	3.10	PE
	-OCH=CHCH_2_	5.90	PLA (ether GPLs)
Fatty acid chains	ω-CH_3_ (methyl) in fatty acyl chains	0.88	
	ω-CH_3_ (methyl) of total omega-3 FA	0.95	
	-(CH_2_)n- (methylene) in fatty acyl chains	1.30	
	-CO-CH_2_-CH_2_- (β-methylene) in the fatty acyl chains	1.59	
	β-CH_2_ (β-methylene) of the sum of AA + EPA	1.67	AA (20:4 ω-6) + EPA (20:5 ω-3)
	-CH_2_-CH= (allylic) in fatty acyl chains	2.04	
	-CO-CH_2_ (α-methylene) in the fatty acyl chains	2.30	Total FA
	α and β CH_2_ (methylene) of DHA	2.38	DHA (22:6 ω-3)
	-CH=CH-CH_2_-CH=CH- of linoleic acid	2.75	LA (18:2 ω-6)
	-(CH=CH-CH_2_-CH=CH)n, n > 1 in the fatty acyl chains	2.80	PUFA
	-CH=CH- in the fatty acyl chains	5.36	UFA

Abbreviations: AA, arachidonic acid; DAGPLs, diacyl glycerophospholipids; DHA, docosahexaenoic acid; EPA, eicosapentaenoic acid; FA, fatty acids; FC, free cholesterol; GPLs, glycerophospholipids; LA, linoleic acid; PC, phosphatidylcholine; PE, phosphatidylethanolamine; PLA, plasmalogens; PUFA, polyunsaturated fatty acids; SLs, sphingolipids; SM, sphingomyelin; UFA, unsaturated fatty acids.

**Table 2:** Demographic, clinical, and biochemical characteristics of the study populations.

				Subgrouping of CHD Patients		
	Patients with NCA (*n* = 46)	Patients with CHD (*n* = 121)	*p*	Mild (*n* = 48)	Moderate (*n* = 36)	Severe (*n* = 37)
**Demographic Parameters**						
Age (years)	59.7 ± 14.0 ^a^	62.5 ± 9.5	NS ^b^	61.8 ± 10.4	62.5 ± 9.1	63.3 ± 9.0
Gender (Males/females)	26/20	70/51		28/20	20/16	22/15
**Risk factors for CHD**						
Hypertension, n (%)	20 (43.5)	65 (53.7)		28 (58.3)	18 (50)	19 (51.4)
Current smokers, n (%)	22 (47.8)	79 (65.3)		34 (70.8)	22 (61.1)	23 (62.2)
BMI (kg/m^2^), n (%)	14 (30.4)	39 (32.2)		15 (31.3)	9 (25)	15 (40.5)
**Biochemical Parameters**						
Total Cholesterol, mg/dL	191.7 ± 36.9	189.1 ± 45.3	NS	188.7 ± 46.2	188.8 ± 49.8	190.1 ± 40.5
Triglycerides, mg/dL	132.5 ± 59.4	137.1 ± 48.6	NS	130.3 ± 43.1	139.6 ± 58.4	143.5 ± 45.0
HDL-C, mg/dL	44.7 ± 10.5	41.7 ± 9.4	NS	42.1 ± 9.6	41.6 ± 8.5	41.3 ± 10.2
LDL-C, mg/dL	120.5 ± 33.5	120.0 ± 40.7	NS	120.5 ± 41.1	119.6 ± 43.9	120.1 ± 37.9
non-HDL-C, mg/dL	147.0 ± 36.0	147.4 ± 43.6	NS	146.6 ± 43.6	147.7 ± 47.0	148.8 ± 41.1
apoAI, mg/dL	122.2 ± 17.3	121.6 ± 19.8	NS	119.6 ± 20.4	124.6 ± 18.4	121.1 ± 20.6
apoB, mg/dL	88.0 ± 21.2	98.4 ± 25.9	NS	92.9 ± 23.4	100.9 ± 27.4	101.9 ± 27.0

NCA, normal coronary arteries; CHD, coronary heart disease. ^a^ expressed as mean ± S.D. ^b^ NS, statistically not significant.

**Table 3:** Red blood cell lipid composition in patients with CHD and those with NCA.

mol/100 mols of Total Lipids	Patients with NCA (n = 46)	Patients with CHD (n = 121)	*p*
***Lipid components***			
**Cholesterol**	**39.37 ± 2.49**	**42.25 ± 2.82**	**<0.001**
**Phospholipids (PLs)**	**58.11 ± 2.70**	**55.61 ± 3.20**	**<0.001**
***Glycerophospholipids (GPLs)***	33.12 ± 2.88	28.35 ± 3.03	<0.001
Phosphatidylcholine (PC) Phosphatidylethanolamine (PE)	15.04 ± 2.64 12.08 ± 1.21	12.10 ± 2.31 11.24 ± 1.73	<0.001 <0.001
Rest GPLs *	6.00 ± 1.73	5.01 ± 1.67	<0.001
***Ether Glycerolipids (Ether GLs)***	9.96 ± 2.03	8.23 ± 2.30	<0.001
Plasmalogens Rest ether GLs	4.49 ± 1.29 5.47 ± 2.09	3.95 ± 1.21 4.28 ± 1.98	<0.05 <0.001
***Sphingolipids (SLs)***	15.03 ± 2.24	19.03 ± 3.09	<0.001
Sphingomyelin (SM) Rest SLs **	14.05 ± 2.31 0.98 ± 0.46	17.64 ± 2.96 1.39 ± 0.94	<0.001 <0.001
**Glycolipids**	**2.52 ± 0.71**	**2.14 ± 1.67**	NS
***Ratios***			
**Cholesterol/PLs**	0.68 ± 0.08	0.76 ± 0.09	<0.001
**SM/PC**	0.98 ± 0.30	1.52 ± 0.42	<0.001
**Cholesterol/PC**	2.72 ± 0.62	3.64 ± 0.85	<0.001
**Cholesterol/SM**	2.87 ± 0.45	2.46 ± 0.44	<0.001
**Cholesterol/PE**	3.29 ± 0.33	3.85 ± 0.66	<0.001
**PE/PC**	0.83 ± 0.18	0.96 ± 0.24	<0.001
**PE/SM**	0.88 ± 0.18	0.66 ± 0.16	<0.001

* Mainly phosphatidylserine, phosphatidylinositol, phosphatidylglycerol, ** mainly ceramide.

**Table 4:** Fatty acid features of RBC membrane phospholipids in patients with CHD and those with NCA.

mol/100 mol of Total Fatty Acids	Patients with NCA (n = 46)	Patients with CHD (n = 121)	*p*
**Saturated Fatty Acids (SFA)**	43.31 ± 6.22	54.31 ± 7.92	<0.001
**Unsaturated Fatty Acids (UFA)**	56.69 ± 6.22	45.69 ± 7.92	<0.001
Monounsaturated (MUFA) Polyunsaturated (PUFA)	3.90 ± 1.73 52.79 ± 6.59	5.55 ± 2.75 40.14 ± 9.17	<0.001 <0.001
**Individual PUFA**			
Linoleic Acid (LA)	10.31 ± 2.15	7.80 ± 1.90	<0.001
Eicosapentaenoic + Arachidonic Acid (EPA + AA)	18.70 ± 4.17	14.43 ± 4.29	<0.001
Docosahexaenoic acid (DHA)	3.44 ± 0.83	2.54 ± 0.63	<0.001
***Ratios***			
**SFA/UFA**	0.79 ± 0.20	1.26 ± 0.45	<0.001
**SFA/PUFA**	0.85 ± 0.23	1.50 ± 0.68	<0.001

All data expressed as expressed as mean ± SD.

## Data Availability

Data will be available upon request.
